# Draft Genome Sequence of Se(IV)-Reducing Bacterium *Pseudomonas migulae* ES3-33

**DOI:** 10.1128/genomeA.00406-15

**Published:** 2015-05-07

**Authors:** Xuanji Li, Witold Kot, Dan Wang, Shixue Zheng, Gejiao Wang, Lars H. Hansen, Christopher Rensing

**Affiliations:** a Department of Plant and Environmental Science, University of Copenhagen, Frederiksberg, Denmark; b Department of Environmental Science, Aarhus University, Roskilde, Denmark; c State Key Laboratory of Agricultural Microbiology, College of Life Science and Technology, Huazhong Agricultural University, Wuhan, China; d Institute of Urban Environment, Chinese Academy of Sciences, Xiamen, China

## Abstract

*Pseudomonas migulae* ES3-33 is a Gram-negative strain that strongly reduces Se(IV) and was isolated from a selenium mining area in Enshi, southwest China. Here we present the draft genome of this strain containing potential genes involved in selenite reduction and a large number of genes encoding resistances to copper and antibiotics.

## GENOME ANNOUNCEMENT

The genus *Pseudomonas* is known to perform diverse tasks, including plant growth promotion, environmental bioremediation, and biological control of various pathogens (1–3). Due to these beneficial properties, the genus *Pseudomonas* has attracted increased scientific interest in recent years. Due to the similarities of the 16S rRNA gene sequences, *Pseudomonas migulae* has been assigned into the *Pseudomonas fluorescens* group (4).

The strain of *Pseudomonas migulae* ES3-33 was isolated from soil samples (soil Se of >20 mg/kg) in an Se mine area in Enshi, southwest China. The 16S rRNA sequences of *Pseudomonas migulae* ES3-33 revealed that it belonged to the species *Pseudomonas migulae*, with sequence similarities of 99.56% using the EzTaxon server (5, 6). Chromosomal DNA was purified from isolates by using a Gentra Puregene Yeast/Bac.Kit (Qiagen). The draft genome sequence of *Pseudomonas migulae* ES3-33 was determined by the Illumina MiSeq 2x250PE platform to generate a paired-end library. *De novo* assembly was performed with SPAdes 3.5.0, resulting in 162 contigs (>200 bp). A total of 5,591 open reading frames (ORFS) were predicted by the RAST server (7, 8) and annotated using the information from GenBank and RAST (9).

The size of the draft genome sequence is 6,075,381 bp, with an average GC content of 59.7%, and the longest contig size assembled is 389,672 bp. The genome consists of 5,404 protein-coding sequences that were assigned predicted functions, 58 tRNA genes, and 10 rRNA genes.

*Pseudomonas migulae* ES3-33 can rapidly reduce selenite to red selenium nanoparticles and is highly selenite resistant, with an MIC of 150 mM. The genome was analyzed and shown to contain potential selenite reductases. Two glutathione reductases (GR), a thioredoxin reductase (THxR), an NADH:flavin oxidoreductase (OYE family), and two nitrite reductases which have previously been reported to reduce selenite were identified on the genome (10–15). An increased presence of copper resistance determinants was observed, possibly due to contamination of the Se mine with other metals. The genome encodes multiple proteins potentially conferring copper resistance, such as the *copABCD* operon also found in *Pseudomonas putida* PNL-MK25 (16), three putative *cus* systems (17), copper-sensing two-component systems (18, 19), two blue copper oxidases, and multiple Cu(I)-translocating P-type ATPases. At least one of the determinants was a mobile element flanked by the Tn7 transposon system.

In addition, *Pseudomonas migulae* ES3-33 also contains genes encoding drug resistance (fluoroquinolones, penicillin, cephalosporin), antibiotic resistance (streptothricin), and other virulence proteins, including multidrug resistance tripartite systems, streptothricin acetyltransferase, fluoroquinolones resistance protein, β-lactamase, class C, and other penicillin-binding proteins.

Multiple virulence genes of *Pseudomonas migulae* ES3-33 might improve our fundamental understanding of multidrug, heavy and transition metal resistance mechanisms, and this knowledge may be applicable to bioremediation.

### Nucleotide sequence accession numbers.

This whole-genome shotgun project has been deposited at DDBJ/EMBL/GenBank under the accession no. JZRI00000000. The version described in this paper is version JZRI01000000.
